# Bacterial Contaminants of Poultry Meat: Sources, Species, and Dynamics

**DOI:** 10.3390/microorganisms5030050

**Published:** 2017-08-25

**Authors:** Amélie Rouger, Odile Tresse, Monique Zagorec

**Affiliations:** Secalim, INRA, LUNAM Université, 44307 Nantes, France; amelie.rouger@gmail.com (A.R.); odile.tresse@oniris-nantes.fr (O.T.)

**Keywords:** chicken meat, bacteria, slaughter, spoilage, pathogen

## Abstract

With the constant increase in poultry meat consumption worldwide and the large variety of poultry meat products and consumer demand, ensuring the microbial safety of poultry carcasses and cuts is essential. In the present review, we address the bacterial contamination of poultry meat from the slaughtering steps to the use-by-date of the products. The different contamination sources are identified. The contaminants occurring in poultry meat cuts and their behavior toward sanitizing treatments or various storage conditions are discussed. A list of the main pathogenic bacteria of concern for the consumer and those responsible for spoilage and waste of poultry meat is established.

## 1. Introduction

Poultry meat consumption is steadily increasing worldwide; the last data available indicate it reached 14.2 kg per capita per year [1]. The developed western countries, particularly the United States of America (USA), are the largest consumers, with 49.8 kg per inhabitant per year [1]. The same trend of consumption increase is observed in the European Union (EU) and in countries of the Organization for Economic Co-operation and Development (OECD). Similarly, poultry meat consumption has doubled in France over the past 30 years and has become the second most consumed meat since 2012, reaching more than 26 kg per capita in 2014 (close to the consumption reported for the EU) after pork meat (32.5 kg per capita) [2]. Among poultry meat products, chicken carcasses, cuts, and processed products are the most consumed (~75% of total poultry meat) followed by turkey (~25%) and, to a lesser extent, duck [3]. In France, 60% of the chicken meat is sold as fresh cuts [2], often stored under various modified atmosphere packaging (MAP) [4]. Vacuum packaging, MAP, chilling, or marinades are different practices for ensuring microbial quality during the storage of poultry cuts, and depend on consumer habits and countries [4,5,6,7]. 

Therefore, ensuring the microbial safety of poultry meat products is an important issue in this context of increasing consumption and production. In fact, during and after slaughtering, the bacteria from animal microbiota, the slaughterhouse environment, and the equipment used contaminate carcasses, their subsequent cuts, and processed meat products. Some of these bacterial contaminants can grow or survive during food processing and storage. The resulting bacterial communities present in poultry meat can include pathogenic species such as *Salmonella* and *Campylobacter*, the two main pathogens responsible for human gastroenteritis due to poultry meat consumption [8]. Since 2005, *Campylobacter* has been the most commonly reported gastrointestinal bacterial pathogen in humans in the EU, where the numbers of reported confirmed cases in 2015 were 229,213 for human campylobacteriosis and 94,625 for human salmonellosis [8]. Poultry consumption has also been shown to be the first cause of foodborne outbreaks in the USA between 1998 and 2012 [9]. Other emerging pathogens, such as *Aeromonas* sp., may also be considered [10]. In addition to foodborne pathogens, bacteria responsible for spoilage may lead to large economic losses. Their growth and metabolic activity during shelf life leading to color, odor, taste, or texture defects are responsible for waste and losses of food products and have therefore an important impact on the economy of the poultry meat production sector. 

Most of the literature dealing with the microbial contamination of poultry meat is dedicated to detecting the presence of pathogens (mainly *Salmonella* and *Campylobacter*) and sometimes to studying their behavior under different decontamination, transformation, or storage conditions. Poultry meat contamination by spoilage bacteria has been less studied and is often limited to their enumeration by counting CFUs (colony forming units) on different, more or less specific, media. Challenge tests, based on the inoculation of individual strains or strain cocktails on meat cuts, have been used to investigate the growth ability of bacteria under various treatments. In contrast, only a few studies have recently used high throughput sequencing technologies to describe poultry meat contaminants, leading to their description at species level [6,7,11,12,13].

The aim of this review is to describe the state of the art regarding the knowledge available on the bacterial contaminants present in fresh poultry meat. The sources of contamination will be listed and the diversity of bacterial contaminants of poultry meat will be presented as well as their behavior along the production process, from the slaughterhouse to the end product, depending on the storage conditions or various treatments.

## 2. Sources of Contamination 

**Figure 1:**
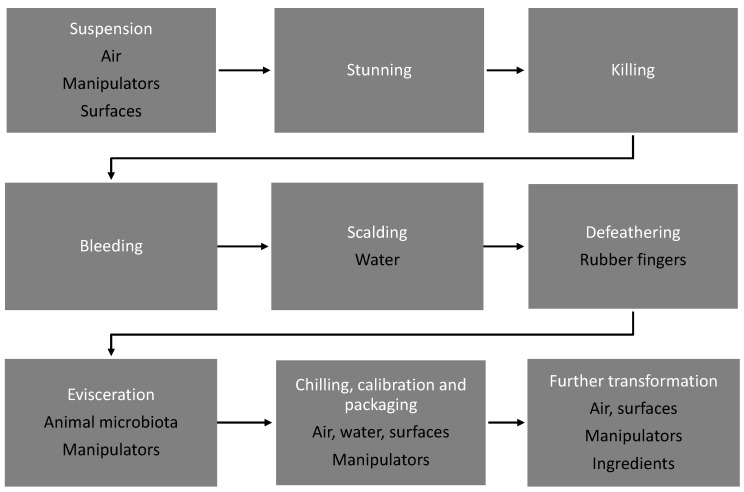
Scheme representing the successive steps from poultry slaughtering to meat production and the associated contamination routes. After transport, the birds are suspended from the conveyor and then stunned and killed. After bleeding, the birds are scalded in hot water at a temperature ranging from 50 to 60 °C to loosen the feathers. Subsequently, the feathers are mechanically abraded from the scalded birds. In large-scale slaughterhouses, feathers are removed using rotating rubber fingers and then the carcasses receive a spray wash prior to evisceration. Evisceration can be carried out by mechanical aspiration or manually after the carcasses have been cut open. At this stage, the gizzard, heart, and liver are also retrieved. Next, the carcasses are chilled, either by immersion in cold water or by air chilling. Subsequent transformation steps include cutting, deboning, grinding, and the use of various treatments for meat product storage such as marinating or addition of different ingredients (salt, spices) in processed products such as sausages.

While muscles are sterile in healthy living birds, various microbiotas are hosted in the digestive tract, lungs, skin, feathers, etc. In slaughterhouses, the surfaces, air (aerosols), and liquids also encompass bacteria. Therefore, carcasses and cuts after animal killing can be contaminated by animal and slaughterhouse environment microbiota. Figure 1 summarizes the different steps in poultry slaughtering and the associated contamination routes. Although there are some differences between the practices in large-scale commercial slaughterhouses and small-scale slaughtering facilities, the main steps of poultry slaughtering are similar [14]. Compared to the slaughtering process of mammals, the main differences to be noted for poultry slaughtering are (*i*) the use of a water bath (hot or chilled) at different stages of the process; (*ii*) the feather removal step, which can be mechanical and is performed differently from removing the skin of mammals; (*iii*) the small size of birds (compared to cattle or sheep, for example) which has consequences on the ease of carcass manipulation and the mechanization of some processes.

During the successive steps described in Figure 1, bacterial contamination may occur from equipment surfaces, water, and animal microbiota. Bacteria from the air and the environment can contaminate broiler meat [15]. The skin of poultry carcasses and cuts is directly in contact with air and equipment surfaces and is therefore easily contaminated. In fresh meat, bacteria are present on the surface rather than in the meat [16]. However, in processed products such as those which have been marinated, bacteria can migrate into the muscles [17]. 

Bacterial contamination by equipment surfaces can take place early in the process. For example, the rubber fingers used for feather removal or conveyor belts can be sources of bacterial contamination [18,19,20]. Even new rubber fingers can host bacteria and be a source of contamination for carcasses [19]. Cross contamination between carcasses or cuts may occur by direct contact or through contact with contaminated surfaces [20]. During the subsequent processing steps (deboning, cutting, mincing, and mixing) for meat-based foodstuff production, manipulators, air and equipment surfaces are the main sources of contamination. In fact, transformation operations increase the surface area of meat in contact with working surfaces and air [21]. Consequently, the level of bacteria is higher in transformed products than on primary cuts [21]. 

The water baths used during the process have a washing effect that diminishes the bacterial loads, but can also promote cross-contamination between carcasses [22,23]. The high temperatures (50 to 60 °C) of the hot water used for scalding contribute to stopping bacterial growth. This helps to diminish the bacterial counts present on skin. However, high temperatures dilate feather follicles and relax poultry skin. Further processing steps may therefore lead to bacteria transfer from feathers to skin and follicles, previously dilated by the hot water, and to entrapping bacteria after the cooling of plucked carcasses. Cold water used for chilling carcasses after evisceration can act as a cross-contamination vehicle between carcasses, but also has a decontaminating effect by rinsing the surface of carcasses, particularly when chlorine is added to the water as in the USA [24]. Although cold water and air chilling procedures have different effects on diminishing *Salmonella* and *Campylobacter* counts, no difference has been observed in the impact of the two procedures on the shelf life of cuts [24]. 

The evisceration step, because of the microbiota present at high counts in the digestive tract, is a critical point of carcass contamination. The gastrointestinal tract of birds hosts many bacteria, including some that can be potentially dangerous for the consumer such as *Campylobacter* spp. or *Salmonella*. There is a correlation between the number of *Campylobacter* in the ceca and the contamination level found on carcasses [25,26]. An average contamination level of 8.05 log CFU/g of ceca and 2.39 log CFU/g of carcasses has been measured [25]. Poultry gut microbiota has been studied in detail, particularly to correlate animal feeding, health, and gut microbiota [27,28,29]. However, to our knowledge, no study has yet been performed to establish a link between the composition of animal microbiota and that of the meat produced from these animals, although it has been reported that bacteria present in meat products originate at least partly from the animal digestive tract [13]. 

The evolution of the level of bacterial contamination throughout the slaughtering process has been described [22,30]. The contamination level of carcasses decreased after evisceration and chilling by immersion in cold water and increased again during storage at refrigerated temperature [30]. Similar results were observed by bacterial enumeration performed on neck skin [22]. This shows the washing effect at different steps, as well as the subsequent bacterial development that can occur during the storage period. After initial contamination, some bacteria can persist during meat product storage and can be recovered from killed animals before the scalding step, as well as after 10 days of the storage of carcasses at refrigerated temperature [30].

## 3. The Major Bacterial Contaminants of Poultry Meat

### 3.1. Bacterial Contaminants

The literature reports different practices for collecting bacteria from cuts or carcasses (rinsing, swabbing, stomaching) and the use of different media or incubation conditions which may lead to different results. Nevertheless, to illustrate the diversity of the bacteria targeted in the literature, we list the results (enumerations in log CFU/g) of several studies carried out by cultural methods on chicken meat (Table 1), resulting in a global inventory of the contaminants that can be encountered. Total viable counts represent various bacterial species, increasing during storage, and varying considerably between samples. As an example, we have previously shown that total viable counts from chicken legs sampled after storage at 4 °C for 2/3rd of their shelf life varied from 3 to 8 log CFU/g [4].

Pseudomonads, often recorded in poultry meat are mainly represented by the species *Pseudomonas fragi*, *Pseudomonas lundensis*, and *Pseudomonas fluorescens* [38,39]. Among *Enterobacteriaceae*, the main genera are *Hafnia* (*Hafnia alvei*, *Hafnia paralvei*), *Serratia* (*Serratia fonticola*, *Serratia grimesii*, *Serratia liquefaciens*, *Serratia proteamaculans,* and *Serratia quinivorans*), *Rahnella*, *Yersinia*, and *Buttiauxella* [40]. Several new *Enterococcus* or *Lactobacillus* species such as *Enterococcus viikkiensis, Enterococcus saigonensis*, and *Lactobacillus oligofermentans* have also been discovered in poultry meat products [41,42,43]. *Brochothrix thermosphacta* has also been often recorded in poultry meat. Among the various reports found in the literature, some targeted more specifically spoilage bacteria whereas others focused on pathogens.

### 3.2. Spoilage Bacteria

Growth of spoilage bacteria lead to defects in the products and can be responsible for unwanted taste, color, odor, texture, or aspect. There are multiple spoilage mechanisms, and they can result from the production of various metabolites such as volatiles or exopolysaccharides. Once bacteria contaminate meat and constitute the initial microbiota, the storage conditions and the various treatments applied shape the fate of this microbiota. The storage temperature as well as the nature and concentration of the gas used in gas mixtures for packaging are selective for some bacterial populations. Storage at low temperature favors the growth of psychrotrophic and psychrophilic bacteria while CO_2_ has an inhibitory effect on *Pseudomonas* spp. Some species can survive throughout the process, such as *Shewanella putrefaciens*, frequently found on carcasses during the slaughtering process and still present after 14 days of storage under air [30]. During storage, the bacterial load increases but the microbiota diversity decreases compared with that initially present [13,44]. Microbial spoilage occurs as a consequence of the growth and metabolic activities of spoiling bacteria. In most studies, the bacteria that dominate spoiled food have been considered those responsible for spoilage and, in some studies, the criterion of microbiological acceptability (total viable counts reaching 7 log CFU/g) has been used to define spoilage [44,45]. Examples of bacteria enumerations in chicken meat products considered as spoiled are listed in Table 2. 

*B. thermosphacta*, lactic acid bacteria (LAB), *Enterobacteriaceae* and *Pseudomonas* spp. are considered potential spoilers of poultry meat. However, from the examples shown in Table 2, the involvement of these bacteria in spoilage could not be clearly concluded. Indeed, the variability in the microbial contaminants present in spoiled poultry meat illustrates the difficulty of clearly identifying the spoilage bacteria. Therefore, the definition of poultry meat spoilage bacteria must be considered carefully.

A list of bacteria present in different meat products and their occurrence depending on the packaging atmosphere used for storage has been established by Doulgeraki and collaborators in 2012 [46]. Some of them were reported as poultry meat spoilage microorganisms. *B. thermosphacta*, *P. fluorescens,* and *S. putrefaciens* are among the spoilage bacterial species most cited in spoiled chicken meat products [23,30,45]. The spoilage potential of *Aeromonas salmonicida*, *P. fluorescens*, *P. fragi* and *S. liquefaciens* has also been evaluated by challenge tests and sensory evaluation [47]. *Aeromonas hydrophila* and *Aeromonas sobria* have been reported as psychrotrophic bacteria that could cause spoilage in addition to being potentially pathogenic for humans [30]. Molecular identification of colonies isolated from marinated spoiled poultry meat showed the involvement of several LAB species; in particular, *Leuconostoc gelidum* subsp. *gasicomitatum* and *Lactobacillus oligofermentans* [6,7,43,48]. Further investigation based on sensory analyses and genome or metabolic activity characterization of these LAB species confirmed their role in spoilage [49,50,51]. MALDI-TOF MS (Matrix-Assisted Laser Desorption/Ionization Time-Of-Flight Mass Spectrometry) was also applied to colonies isolated from chicken breasts stored under two different MAPs and at two different temperatures in order to identify spoilage bacteria [44]. *B. thermosphacta*, *H. alvei* and bacteria belonging to the genera *Carnobacterium, Janthinobacterium, Pseudomonas*, and *Serratia* were identified among the dominant contaminants. However, in this study, spoilage was considered to occur when total viable counts reached 7 log CFU/g, with no indication about sensory deterioration [44]. 

### 3.3. Pathogens

Numerous articles have investigated the prevalence of various pathogens in poultry meat. Among these, *Campylobacter* and *Salmonella* make up a large majority of the reports. These two human pathogens can be present at high loads in the gastrointestinal tract of birds but, after contamination of poultry meat, it is important to detect their presence even at a very low level. Therefore, some studies have focused on establishing correlations between the occurrence in animals and in meat [25]. The emergence of antimicrobial resistance among foodborne pathogens is also extensively recorded [52]. In addition, the impact of breeding or farming on the prevalence and antibio-resistance in *Campylobacter* has been addressed [53]. Methods for the fast and accurate detection and identification of *Campylobacter* have been proposed [54]. Nevertheless, the data obtained by different methods should be carefully interpreted. As an example, the *Campylobacter* proportion enumerated in poultry feces determined either by high-throughput sequencing or by plating on various *Campylobacter* selective media gave quite different values [55]. Both *Campylobacter jejuni* and *Campylobacter coli* can be isolated from poultry meat [25], but also from human clinical cases that may result from contaminated food consumption [56]. No clear correlation could be established between the presence of *Campylobacter* in poultry meat and the level of bacterial contamination of chicken or turkey cuts [54]. *Salmonella enterica* is among the most tracked human pathogen, with the serovar Enteritidis being mainly associated with poultry meat and with outbreaks [57]. Other foodborne human pathogens present in various meat products have also been investigated, such as *Listeria monocytogenes* [58,59,60,61,62]. *Listeria* spp. prevalence in poultry meat is noticeable, with *Listeria innocua* as the dominant species followed by *L. monocytogenes* and several other *Listeria* species (*Listeria welshimeri*, *Listeria grayi*, and *Listeria ivanovii*). The prevalence of *Staphylococcus aureus* on poultry meat products has been addressed, although most of the literature has focused on antibiotic resistance and typing of the isolates [63,64,65]. Although there are a few reports on the detection of *Clostridium perfringens* on poultry meat [61], most of the literature focuses on assessing and modeling its growth on meat after spore germination following the slaughtering process [66,67,68]. Lastly, the emergence of *Aeromonas* from poultry meat products as a vector of human infection has also been reported [10]. Among *Aeromonas* spp. detected on poultry carcasses, *Aeromonas caviae*, *A. hydrophila*, *Aeromonas salmonicida-masoucida*, and *Aeromonas schuberti* have been reported to survive after 14 days of product storage [30].

## 4. Variability of Bacterial Communities Regarding Different Matrices and Processes 

We have combined the data reported in the literature to draw a picture of the bacterial contaminants occurring in poultry meat depending on different variables. We chose to select variations depending on the meat matrix or on the storage/transformation process. The bacterial contaminants present in poultry carcasses and cuts and their growth depend on different factors: the storage temperature, the gas composition used for MAP, the composition of marinades or various chemical treatments that can be applied to control bacteria. A number of studies were selected to illustrate the diversity of the methods used.

### 4.1. Variability of Bacterial Contaminants Regarding Meat Matrix and Origin

Most of the literature focuses on chicken meat and, to a lesser extent, turkey meat. A comparative study of the microbiological quality of poultry meat in Morocco showed that turkey meat was more contaminated (5.4−7.4 log CFU/g total aerobic counts) than chicken meat (4.5−6.6 log CFU/g) [61]. Nevertheless, for several pathogens (*Escherichia coli*, *S. aureus*, and *C. perfringens*) the contamination level was similar in chicken and turkey meat. The difference might result from the different farming conditions and/or intrinsic differences between these two birds. These authors also noticed that the traditional slaughtering process increases contamination by microbial communities. This correlates with another observation, which reported a higher contamination level of skins of chicken carcasses from traditional markets and artisanal slaughterhouses [33]. This study, also carried out in Morocco, showed higher counts of mesophilic and psychrotrophic bacteria, total and fecal coliforms, and *S. aureus* on artisanal products than on carcasses purchased from supermarkets. 

The contamination level regarding different cuts or raw *vs.* transformed products has also been evaluated. The mesophilic bacteria from various poultry cuts (thighs, wings, giblets, hamburgers, and sausages) were enumerated These were higher in processed products (hamburgers, sausages) with approximately 7 log CFU/g, than in the fresh cuts (thighs, wings) with approximately 5.7 log CFU/g [21]. This may result from the temperature during the transformation process (10 °C) and from the mixing steps that increase the surface area of meat in contact with surfaces and air, both favorable to bacterial growth and to the possibility of increased contamination.

### 4.2. Variability of Bacterial Contaminants Regarding Storage Temperature 

The importance of temperature for bacterial growth can be assessed at different critical points between the slaughtering and the consumption of the product, in particular (i) during carcass handling (the temperature in the processing plants is usually about 10 °C); and (ii) during the storage of meat products (with an estimation of a rupture in the cold chain between the time of sale and the consumer’s fridge, whose temperature is estimated to be higher than 4 °C).

The effect of chilling carcasses using chilled air or a cold water-bath on their microbial contaminants has been assessed [69]. Refrigeration by chilled air slows down the development of the total viable count (approximately 1 log) and causes a rapid decrease in temperature. This inhibits the multiplication of *Salmonella* and *Campylobacter*, thus chilled-air cooling would be more efficient. However, it is necessary to take the fact that *Listeria* can grow at this storage temperature into account.

The product shelf life can be increased by storage at low temperature and the absence of a break in the cold chain [70]. The shelf life can even be doubled when the temperature is lowered to 3.4 °C compared to storage at 8.3 °C. Low temperatures delay the growth of *Enterobacteriaceae*, which can produce sulfuric compounds and the organoleptic deterioration of the meat quality. On the other hand, the growth of psychrotrophic bacteria is enhanced; at 4 and 7 °C, the total viable counts develop faster than at 0 °C [69]. A faster development of total viable counts is also observed at 10 °C when compared to 4 °C [45]. Storage at 4 °C is damaging for *B. thermosphacta* and *S. putrefaciens* growth after 7, 10 or 14 days whereas *A. hydrophila* and *A. sobria* are psychrotrophic bacteria that can develop at low temperature [30]. Smolander et al. (2004) also pointed out, however, that the shelf life of products cannot be lengthened too much by storage at 0 °C, because pathogenic agents such as *Listeria* can multiply at these temperatures [70]. 

### 4.3. Variability of Bacterial Contaminants Regarding the Packaging Gas Composition 

Dioxygen-enriched atmospheres have been largely used for ensuring typical red meat color during storage. However, poultry cuts may be considered differently [71] and different gas compositions are in use for poultry cuts [4,71]. CO_2_ has a bacteriostatic effect, which inhibits the growth of aerobic microorganisms such as *Pseudomonas* spp. that are considered putative spoilage organisms. The effect of different gas compositions on microbial contaminants has been evaluated [35,72]. As shown in Table 3, the storage period to reach spoilage (estimated as the time for total viable counts to exceed 7 log CFU/g) can be prolonged by CO_2_-enriched atmospheres when compared to storage under air. The shelf life before spoilage occurrence can be extended from six days under air to 12 and 15 days under MAP with 30% CO_2_–70% N_2_ and 70% CO_2_–30% N_2_, respectively [35] or from 5 to 8 days with 30% CO_2_−70% N_2_ [72]. *B. thermosphacta, Pseudomonas*, and *Enterobacteriaceae* counts did not seem significantly affected by the type of packaging but were detected as bacteria responsible for spoilage [32]. LAB counts varied depending on gas composition, but also between the studies [35,72]. Prolonged shelf life by CO_2_ enriched atmospheres (30% CO_2_–70% N_2_, 60% CO_2_–40% N_2_, and 90% CO_2_–10% N_2_) has also been reported for precooked chicken breasts [73].

Replacing nitrogen with argon in the composition of MAP (proportion from 15% to 82%) was tested [74]. No strong difference was observed, with only *B. thermosphacta* appearing to be significantly affected by a high proportion of Ar in the gas mixture. Nevertheless, the various proportions of Ar or N_2_/CO_2_/O_2_ in the gas mixtures tested shaped differently the growth dynamics and the ratio of different populations (LAB, *B. thermosphacta*, *Pseudomonas* spp., and *Enterobacteriaceae*). The growth of mesophilic LAB was favored by anaerobic conditions, high quantities of CO_2_, or both. At low Ar or N_2_ concentration (15%), the dominant bacteria were *Pseudomonas* spp., *Enterobacteriaceae*, and *B. thermosphacta* with dominance of the latter increasing during storage [74]. These authors noted the ability of *Pseudomonas* spp., considered aerobic bacteria, to grow with only residual amounts of O_2_. 

A pretreatment of 3 hours with 100% CO_2_ prior to packaging under 70% CO_2_–15% O_2_–15% N_2_ improved the microbiological quality of the meat of raw chicken drumsticks and prolonged shelf life [31]. The *Pseudomonas* counts, as well as the total aerobic counts, were significantly lower after 7 and 12 days of storage when a CO_2_ pretreatment was applied. Such treatment had no additional effect on coliforms, which were undetectable after seven days of storage under MAP, whether or not a CO_2_ pretreatment was applied. Such an effect on the shelf life resulted from a better availability of CO_2_ in the headspace during storage because of the dissolution of CO_2_ in meat after the pretreatment.

Although varying gas composition of MAP certainly acts on shelf life and spoilage of poultry cuts, no clear description on the effect of gas on the growth of bacterial at the level species has been reported. 

### 4.4. Variability of Bacterial Contaminants in Marinated Chicken and with Various Additives

The definition of marinade varies according to the country [46,75]. Marinades may be composed of a mixture of oil or salt and phosphates (in France and Spain, for instance) or a sauce with oil, organic acids, or spices, essential oil and thickener (Finland, China, and Italy). In all cases, marinades are associated with storage under different MAPs.

The combination of oregano essential oil addition at 0.1% or 1% with MAP on the microbiological quality of chicken cuts has been studied [35]. The addition of 0.1% oregano essential oil increased the shelf life by 3–4 days, while the increase provided by the gas mixture (70% CO_2_−30% N_2_) was only 2–3 days. The combination of a marinade with oregano essential oil and storage under MAP showed that the two treatments could be added as the shelf life reached more than 20 days with a decrease in the total viable count of 2–3 log CFU/g [35].

In Finland, the consumption of marinated poultry products packaged under MAP is common and the effect of the marinade on their microbial safety has been well documented. The Finnish marinade can be complex as it is composed of acetic acid, honey, glucose, maltodextrin, NaCl, phosphate, rape seed oil, spices (sweet pepper, curry, black pepper, garlic and turmeric), thickener (guar gum and xanthan gum), and yeast extract [7]. Such marinades may influence the LAB population by favoring the growth of specific species, particularly because of the source of carbohydrates they provide [46]. The MAP commonly used in Finland is composed of 65% N_2_ and 35% CO_2_. The marinade favors a LAB psychrotrophic population, not detected in the unmarinated products [46]; especially *L. gasicomitatum*, also present in spoiled meat and seafood products [13] and in some vegetables associated with marinated fish products [76]. This bacterial species, unable to survive in the digestive tract of the animal, certainly originates from the environment and is adapted to the cold because it can persist throughout the transformation process [46]. As the combination of MAP and marinade favors the emergence of this group of bacteria, it is necessary to understand their mechanism of adaptation to monitor them in such products. It should be noted that the marinade had no effect on *Campylobacter* [46]. In a study combining the identification of isolates, as well as 16S rDNA gene pyrosequencing and metagenomics an overview of the effect of marinades on broiler fillet strip microbiology was reported [6]. Samples stored at 6 °C under MAP (65% N_2_−35% CO_2_) with and without marinade were compared. The combination of cultural and molecular methods confirmed that among LAB, marinade favored *Leuconostoc* and particularly *L. gasicomitatum*, and decreased *B. thermosphacta*, *Clostridium* spp., and *Enterobacteriaceae*. Among LAB belonging to the genus *Carnobacterium*, *C. divergens* was present in higher amounts than *C. maltaromaticum*, although both species seemed sensitive to marinade, certainly because of the presence of acetic acid.

### 4.5. Variability of Bacterial Contaminants Regarding Sanitizing Treatments

The effect of several sanitizing treatments tested on in laboratory media or on meat cuts has also been assessed. These treatments are summarized in Table 4.

In laboratory conditions (in vitro), the effect of three treatments on the lag phase and on the maximum growth rate was measured on several pathogenic (*S. enterica* serotype Enteritidis, *L. monocytogenes*) and spoilage (*P. fluorescens* and *B. thermosphacta*) bacteria [78]. Acidified sodium chlorite was the most effective at decreasing the growth of all tested bacteria, whereas trisodium phosphate and citric acid were more effective against Gram-negative and Gram-positive bacteria, respectively. However, the effectiveness varied with the concentrations used. For example, at low concentrations trisodium phosphate increased the growth rate of *S. enterica* and *L. monocytogenes*. As well as the consequence of the strong effect of citric acid toward *B. thermosphacta*, the possible increased growth of pathogens was questioned. Thus, the authors questioned the potential danger to consumers of some treatments, by increasing the proportion of pathogenic bacteria with regard to the spoilage ones. The same conclusion about the dangerous effects of treatments favoring pathogenic bacteria as an indirect consequence of inhibiting spoilage bacteria was shared by other authors [80].

In addition, the acid stress response of *L. monocytogenes* after exposure to acidic poultry meat decontaminants may even enhance its survival of a subsequent exposure to stronger acidity, such as that encountered during gastric transit [79]. This adaptation to acidic conditions involves membrane fluidity in *L. monocytogenes* and *S. enterica* and suggests that other decontaminants should be preferred rather than sub-inhibitory concentrations of citric acid or peroxy acids [77]. In summary, these studies showed that trisodium phosphate and citric acid were effective against Gram-positive pathogenic bacteria and peroxy acids and acidified sodium chlorite against Gram-negative bacteria. However, the observation of significant reductions in the microbial level immediately after treatment resulted from trials that were not performed in real meat conditions.

Naturally contaminated meat matrices have also been used [36,37,84,85,86]. In these conditions, all decontaminants tested (trisodium phosphate, lactic and citric acids, peroxy acids, acidified sodium chlorite) reduced the total viable counts, *Enterobacteriaceae*, *Pseudomonas,* and LAB counts. The most effective concentrations reported were 14% for trisodium phosphate and 5% for citric acid. Trisodium phosphate, citric acid, acidified sodium chlorite, and peroxy acids were considered interesting treatments for extending the shelf life and improving the safety of products [37]. 

The effectiveness of chemical decontaminants and physical treatments (such as steam, hot water, and electricity) during or after the slaughtering process has been reviewed [87]. These authors emphasized that besides the relative effectiveness of treatments toward a variety of bacterial species, these must be considered as part of an integral food safety system. In that sense, some authors also completed the analysis of treatments of carcasses against pathogens with a sensory analysis performed by trained panelists on the cooked carcasses [85]. Since then, several other authors have also included the analysis of the sensory impact of decontamination treatments [88].

The impact of other physical decontamination processes on the microbiology of poultry meat has also been investigated. High hydrostatic pressure associated with the addition of nisin or glucono delta-lactone was effective at decreasing the counts of psychrotrophic bacteria and, to a lesser extent, mesophilic bacteria [89]. Gamma irradiation associated with storage under different MAPs was also effective at reducing LAB, *B. thermosphacta*, *Pseudomonas,* and *Enterobacteriaceae* [90]. Nevertheless, although such physical treatments have proven their ability to reduce the microbial load, they may have indirect effects on the sensory attributes of meat (color, texture). In addition, the perception by consumers of such practices can be controversial and their use is regulated differently depending on the country [91,92].

## 5. Conclusions 

The poultry meat sector tends to provide ready to eat products, which are safe for the consumer and have a long shelf life. The biological hazards associated with poultry meat production and consumption have been well identified, ranking *Campylobacter* spp. and *Salmonella* spp. as a high risk [93]. Such ranking took the severity of the illnesses caused by these pathogens, their impact on human health, the number of cases, and the occurrence of the risk in the poultry meat production chain into account. Consequently, the impact of various treatments (temperature, chemical treatment, marinade, or preservation processes) in reducing pathogens has been investigated. Many studies have also been conducted to test such treatments for extending the shelf life and avoiding spoilage.

The large number of publications dedicated to poultry meat microbiology and the variety of the results highlight the wide diversity of the microbiological status of poultry meat products. The bacterial loads can vary by several log CFU/g for similar cuts, stored under similar conditions. To date, the microbial ecology of poultry meat products has been considered mainly through cultural methods, which can introduce a bias because of the relative selectivity of the media used. In particular, poorly selective media targeting large families of bacteria such as LAB or *Enterobacteriaceae* have been used, leading to a poor characterization of the bacterial species present. The studies aiming at assessing the spoilage and/or shelf life of the products have used various criteria that make it difficult to describe clearly which bacteria can spoil poultry meat under which conditions, except for marinated poultry. In fact, marinades providing sugar and acetic acid lead to a pressure selection on the bacterial diversity, including bacteria responsible for spoilage, with the identification of the bacterial functions involved in spoilage appearance. Concerning pathogens, most of the efforts have focused on tracking them, while only a few describe their behavior in the meat matrix and consider the meat microbiota. In fact, two approaches can be distinguished: one focusing on only one or a few species, mostly pathogenic, with little attention paid to the microbiota because of the low contamination level of pathogens regarding that of total counts; and one focusing on a wider range of microbes, but assessing microbiota with techniques that induce a bias in the identification or that are generalist because of the media used. A third approach, already used for investigating complex environments, has recently appeared in food microbiology and tends to study the microbiota by non-cultural methods. The advantage of the latter is a better description of the bacterial species present in poultry meat, regardless of the detection of pathogens that are often present at a lower level. Finally, although the gastrointestinal tract of birds and slaughtering facilities have been identified as the main reservoirs for the origin of poultry meat contaminants, there is little knowledge about the flux of microbiota along the production chain from the animals to the end products. Indeed, the few studies about the transmission from animal to meat have mainly focused on pathogens. 

The combination of high throughput sequencing approaches with highly selective cultural methods throughout the production chain will be necessary to assess the sources of meat contaminants, their identification, and their dynamics during processing and storage. Moreover, metatranscriptomics may also be useful for determining the metabolic functions expressed by bacterial contaminants during meat processing and storage. Combining this with metabolomics should unravel the complex behavior of the poultry meat contaminants along the chain of food production. This should help to better manage meat ecosystems and improve the microbial quality and safety of food. 

## Figures and Tables

**Table 1:** Values reported for various bacterial groups or species contaminating poultry meat at the beginning of the storage period. Values are expressed in log colony forming units (CFU)/g and have been collected from different reports.

Reference ^1^	[31]	[32]	[33]	[34]	[35]	[36]	[37]
Total viable count	5	4.9	ND ^2^	4.88−5.41	4.28	5.66	ND
Psychrotrophic bacteria	ND	ND	4.02−4.48	ND	ND	ND	4.34
Mesophilic bacteria	ND	ND	4.74−6.18	ND	ND	ND	5.10
LAB ^3^	ND	3.9	ND	ND	3.66	ND	3.50
*Pseudomonas*	3.5	4.2	ND	ND	3.38	ND	4.70
*Enterobacteriaceae*	ND	ND	ND	2.58−3.53	ND	ND	2.78
*B. thermosphacta*	ND	3	ND	ND	3.04	ND	4.06
*E. coli*	2	ND	0.70−2.34	2.60−3.63	ND	ND	ND
Coliforms	2.2	ND	3.54−4.64	ND	ND	3.08	2.86
*S. aureus*	ND	ND	0.68−2.43	ND	ND	ND	ND

^1^ Data were collected from references [31,32,33,34,35,36,37]; ^2^ ND: not determined by the authors; ^3^ Lactic acid bacteria.

**Table 2:** Enumeration of bacteria from spoiled chicken meat. Storage conditions and values of bacterial counts have been collected from different reports.

Storage conditions	[45] ^1^	[36]	[35]	[31]	[32]	[46] ^2^
Duration (days)	4	5	9	11	15	Until spoiled
Temperature (°C)	4 to 10	7	4	3	4	6
Packaging	Air	Air	Air	70% CO_2_, 15% O_2_, 15% N_2_	Air	65% N_2_, 35% CO_2_
Total viable count ^3^	7.55	8.27	7.55	6.5	8	9.14
LAB	8.04	ND ^4^	7.02	ND	7	9.04
*Enterobacteriaceae*	8.36	ND	ND	ND	6	7.59
*B. thermosphacta*	ND	ND	7.23	ND	7	ND
*Pseudomonas*	6	ND	7.21	5	6	ND
Coliforms	ND	4.38	ND	3.7	ND	ND

^1^ Data were collected from references [31,32,35,36,45,46]; ^2^ Marinated poultry; ^3^ Bacterial counts are expressed as (log CFU/g), except in A (log CFU/cm^2^); ^4^ ND: not determined.

**Table 3:** Comparison of bacterial counts at spoilage time after storage under air or under two different MAPs.

	**Days to Reach Spoilage (Total Viable Count ≥ 7 log CFU/g)**				
	Air	Air	30% CO_2_−70% N_2_	30% CO_2_−70% N_2_	70% CO_2_ 30% N_2_
	6 ^1^	5 ^2^	12 ^1^	8 ^2^	15 ^1^
	**Bacterial Counts (log CFU/g) When Spoilage Was Reached**				
LAB	2.91	3.2	6.88	4.1	6.89
*Pseudomonas*	6.28	6.2	6.83	6.1	6.71
*B. thermosphacta*	6.90	ND ^3^	6.39	ND	7.21
*Enterobacteriaceae*	6.15	5.8	6.42	5.8	6.71

^1^ Data were collected from [35]; ^2^ Data were collected from [72]; ^3^ ND: not determined.

**Table 4:** Examples of sanitizing treatments tested and experimental designs.

Experimental Design	TSP ^1^	ASC	CA	PA	CD	LA	AA	KP	KO	G
Laboratory conditions										
[77] ^2^	X ^3^	X	X	X						
[78]	X	X	X							
[79]	X	X	X	X	X					
Challenge-tests										
[80]	X	X	X	X	X					
[81]	X	X	X	X	X					
[82]	X	X	X	X						
[83]	X									
Natural contamination										
[37]	X	X	X	X						
[84]								X	X	
[85]	X					X				X
[86]	X	X	X	X		X				
[36]	X	X	X				X			

^1^ TSP: trisodium phosphate, ASC: acidified sodium chlorite, CA: citric acid, PA: peroxy acids, CD: chlorine dioxide, LA: lactic acid, AA: acetic acid, KP: K_3_PO_4_, KO: potassium oleate, G: glutamal; ^2^ Examples were collected from references [36,37,77,78,79,80,81,82,83,84,85,86]; ^3^ Compound analyzed in the corresponding article.
